# Obstetric interventionism and maternalization ofwomen in childbirth care in Uruguay, 1920-1940

**DOI:** 10.1590/S0104-59702024000100025

**Published:** 2024-06-17

**Authors:** Natalia Magnone Alemán

**Affiliations:** i Profesora adjunta, Departamento de Trabajo Social/Facultad de Ciencias Sociales/Universidad de la República. Montevideo – Uruguay natalia.magnone@cienciassociales.edu.uy

**Keywords:** Medicalization, Maternity, Birth, Intervention, Uruguay, Medicalización, Maternidad, Parto, Intervención, Uruguay

## Abstract

This article analyzes the speeches of leading doctors in the creation of the specialty in childbirth care: gynecotology. Between 1920 and 1940, under the influence of eugenic and maternalist thinking, in a context of valuing the well-being of children, medicine built a new obstetric interventionism under the foundation of improving fetal viability. The supposed female “maternal instinct” was, thus, appealed to improve acceptance of the medical mandate. At the same time, doctors recognized their difficulties in providing adequate care. They did not wait long enough and tended to intervene in unnecessary physiological processes.

## Resumen

En este artículo se analizan discursos de médicos de referencia en la creación de la especialidad en la atención del parto: la ginecotocología. Entre 1920 y 1940, bajo la influencia del pensamiento eugenésico y maternalista, en un contexto de valorización del bienestar de la infancia, la medicina construyó un nuevo intervencionismo obstétrico bajo el fundamento de mejorar la viabilidad fetal. En ese marco se apeló al supuesto “instinto maternal” femenino para mejorar la aceptación del mandato médico. A la vez, los médicos reconocieron sus dificultades para asistir de forma adecuada. No esperaban el tiempo suficiente y tendían a intervenir más de lo necesario en procesos fisiológicos que no lo requerían.

En las primeras décadas del siglo XX, Uruguay transitaba en un proceso de construcción de su estado de bienestar, lo que redundó en la mejora de la infraestructura, la vivienda, el saneamiento, la salud, los derechos del trabajo y la escolarización, entre otros asuntos (Birn, 2019). En ese marco, como ha sido relevado para varios países, los estados erigieron políticas públicas para promover el cuidado de la infancia, lo que implicaba transformar las formas de crianza anteriores y mejorar los índices de mortalidad infantil (Peruchena, 2010, 2020; Sapriza, 2001; Nari, 2004; Nash, 1991; Tarducci, 2013; Birn, Pollero, Cabella, 2003). En el año 1900, Uruguay registraba una de las tasas de natalidad más bajas del mundo (93 por mil). El problema apareció a posteriori, debido a su estancamiento por cuatro décadas consecutivas (Birn, Pollero, Cabella, 2003; Leopold, 2002). Los expertos en salud pública propusieron un conjunto de medidas (siguiendo el modelo francés) que incluían la construcción de un hospital infantil y distintas políticas de apoyo materno (Birn, 2019). Similar a procesos de la región (Zárate, González, 2018), se instauraron maternidades, refugios para embarazadas, consultorios de Gota de Leche^1^ y otros servicios atinentes a lo legal-social de la maternidad (Birn, 2019). La medicalización del parto se ubica en este contexto.

En la región existen varios estudios que vienen dando cuenta de las particularidades de los procesos de institucionalización y medicalización del parto. En Argentina, Marcela Nari estudió las ideas y prácticas en torno a la maternidad en la ciudad de Buenos Aires entre 1890 y 1940, y encontró que hubo influencia del discurso de la ciencia médica y eugenésica en la conformación de un ideal de mujer vinculada a la reproducción. Según la autora, la maternalización fue parte de un conjunto de transformaciones económicas, sociales y políticas, en el marco de sociedades capitalistas, que pretendían cambiar sus pautas poblacionales, colocando a la maternidad como asunto político. La maternalización no implicaba la obviedad de que las mujeres podían convertirse en madres, sino que “solo debían ser madres. Cualquier otra actividad, deseo, sentimiento, ponía en peligro su función maternal” (Nari, 2004, p.101). Años después, Ianina Lois (2018) estudió los discursos e imágenes en torno a la maternidad implicada en las intervenciones ante el embarazo, parto y puerperio por parte de grupos médicos en Buenos Aires entre 1900 y 1920. Su investigación registra el proceso de construcción de control médico y masculino de la reproducción, así como la nueva exigencia del parto en el hospital, donde el embarazo pasó a concebirse como un proceso patológico que requería “la presencia del médico” (p.230). En Brasil, Marivaldo Cruz de Amaral (2008, p.928) analizó el papel de la prensa de Bahía en la medicalización del parto y concluyó que la prensa fue utilizada como estrategia de construcción de consensos y disensos, así como de opiniones en torno a la hospitalización del nacimiento. Lo que da cuenta que el proceso de conducción de las mujeres hacia el parto hospitalario precisó de distintos soportes sociales. Aline Medeiros (2013) se enfocó en la dinámica hospitalar de la Maternidade Dr. João Moreira, en Fortaleza, en las primeras décadas del siglo XX. Allí identificó que los médicos visualizaron al cuerpo femenino como peligroso para el feto, lo que ofició de fundamento para la legitimación de la asistencia médica del parto. Por su parte, Ana Vosne Martins (2004) estudió discursos médicos en Brasil y Europa y dio cuenta de la relación entre la especialización médica en el parto y la construcción de la pasividad femenina en la atención. En Perú, Jorge Lossio, Ruth Iguiñiz-Romero y Pilar Robledo (2018) ubicaron a la medicalización del parto como parte de un proceso más amplio de construcción de un cierto tipo de Estado y de patriarcado común en la región.

Las investigaciones señaladas advierten que, a la vez que se iba mejorando el acceso a servicios y tecnologías obstétricas – con su correlato en la mejora de la morbimortalidad materno-infantil –, el modelo de atención en construcción propiciaba relaciones de subordinación de las mujeres hacia el poder médico. Desde visiones del cuerpo femenino como patológico y peligroso para el feto, se fue legitimando la hegemonía médica en el control del parto (Montes, 2008; Lois, 2018).

Por su parte, la historiografía uruguaya ha dado cuenta de la centralidad de la maternidad para las políticas públicas de las primeras décadas del siglo XX (Sapriza, 2001; Cuadro, 2016; Peruchena, 2010, 2020), así como también ha relevado el proceso de construcción de hegemonía de la medicina en la atención de la salud reproductiva (Barrán, 1999, 2008; Sapriza, 1996; Collazo, Palumbo, Sosa, 2012). El presente artículo, aporta a la historia de la medicalización del parto en el país, enfocándose en las razones por las cuales el poder médico fue construyendo legitimidad para asistirlo, así como también, analiza la relación entre las intervenciones obstétricas de la nueva especialización y la maternalización de las mujeres. Para ello, se analizán discursos de médicos referentes en la atención del parto en momentos de la creación y consolidación de la especialización en obstetricia. El análisis de estos discursos permite acceder a saberes y valores de la medicina sobre el embarazo, parto y puerperio en épocas de instalación del modelo médico de atención (Barreto, 2008). Así como también, acercarnos a las ideas sobre las mujeres, la maternidad y la intervención obstétrica de aquel conocimiento autoritario y autorizado (Jordan, 1997).

La metodología utilizada se sustenta en un acercamiento histórico que integra técnicas de análisis de otras ciencias sociales, opciones teórico-metodológicas acordes con el entrelazamiento de la investigación social e histórica (Vommero, 2021, p.99). El corpus reúne una selección de textos, con base en un criterio teórico, de forma de abordar las concepciones de los médicos acerca del parto, de las intervenciones recomendadas, de las profesiones idóneas para su atención, de las preocupaciones en torno a la maternidad y de la relación entre ellos y las mujeres asistidas. Se realiza análisis de contenido de conferencias y artículos de médicos de referencia en la etapa de consolidación de la especialización en la atención del parto (entre 1920 y 1940). En esos años, se consolidó un cambio de enfoque que postuló la unión de la ginecología con la obstetricia, fundando la ginecotocología.^2^ El material analizado se compone de 13 documentos del médico uruguayo Augusto Turenne (1870-1948), quien ocupa un lugar preferencial en la historia de la obstetricia uruguaya (Pou Ferrari, Pons, 2012, p.122). Fue el médico que realizó “una campaña sistemática a lo largo del siglo, para lograr la medicalización del parto” (Sapriza, 2002, p.94). Además, ofició de decano de la Facultad de Medicina y fundó, en el año 1915, la primera maternidad del país. Creador de la obstetricia social (Mañé Garzón, 1996, p.132), fue uno de los médicos de mayor prestigio en el medio científico y político en las primeras décadas del siglo XX (Birn, Pollero, Cabella, 2003). Se integran también, dos obras del médico uruguayo Juan Pou Orfila (1876-1947), precursor de la unión entre la ginecología y obstetricia y autor de varias publicaciones de referencia (Pou Ferrari, Pons, 2012) y dos trabajos del ginecólogo argentino Alberto Peralta Ramos (1880-1954), quien fue fundador y director del Instituto de Maternidad de Buenos Aires.

## El contexto de la asistencia al parto en la primera mitad del siglo XX

En pleno proceso de reforma social y moral (Barrán, 2008), el Estado uruguayo fue dando respuesta a distintos problemas sociales. En lo que refiere a los asuntos materno-infantil, se registró una apuesta muy importante por los cuidados de la salud y los derechos de los niños. La preocupación por el bienestar de la infancia abarcó amplios espectros, incluso convocó a las feministas, que organizaron congresos, donde reformadores sociales, médicos, abogados y profesionales discutieron acciones para mejorar la calidad de vida de niños y mujeres de las clases más empobrecidas (Birn, 2019).

El marcado descenso de la natalidad, el estancamiento de la mortalidad infantil y la elevada proporción de abortos, alimentaban la preocupación por “dignificar” la figura materna y mejorar así la situaciòn de la infancia (Rodríguez, Sapriza, 1984, p.10). Los preceptos eugenistas e higienistas formaron parte de los discursos de los referentes de la política de salud de la época y fueron guía en la construcción de los significados modernos tanto de la maternidad como de la infancia. Recordemos que en el Montevideo de la segunda mitad del siglo XIX ya se publicaban obras centradas en educar a “las jóvenes e inexpertas madres, en la ciencia maternal” (Osta, Espiga, 2018, p.107). El niño, pasará de ser concebido como un adulto en pequeño “a ser visto como un ser diferente con derechos y deberes propios de su edad” (Barrán, 2008, p.298). Si el bienestar infantil se sacralizaba (Villalta, Gesteira, Graziano, 2019), su mortalidad se transformaba en una gran preocupación. Los datos estremecían. En 1924, el 10% de los nacidos vivos fue depositado en el torno,^3^ y casi el 40% de los que fueron llevados a las casas cuna murieron antes del año (Morquio citado en Birn, Pollero, Cabella, 2003). El médico Julio Bauzá, de reconocido trabajo en pediatría, agregó tres causas de mortalidad a las cinco previamente identificadas (el clima, la higiene, factores sociales, epidemias y falta de asistencia médica). Las nuevas fueron: abandono materno, ignorancia de las madres pobres sobre higiene alimentaria y escasa vigilancia domiciliaria de las mujeres inscriptas en los consultorios Gota de Leche. De este modo, las mujeres madres de sectores pobres comenzaron a ser vigiladas por el Estado y a ser encauzadas hacia los distintos servicios maternales antes esbozados (Birn, Pollero, Cabella, 2003).

Tal como se ha documentado para el caso argentino (Nari, 2004), los médicos uruguayos comenzaban a rechazar la crianza en hospicios y la alimentación con nodrizas domésticas, lo cual implicó un fuerte adoctrinamiento hacia las mujeres para que dieran de mamar a sus hijos e hijas. El primer consultorio de Gota de Leche del Uruguay exhibía en la pared la siguiente frase: “El único alimento racional del recién nacido es el que mana del pecho de la madre” (Uruguay, 1913, p.56). El mandato de lactancia constituyó una parte importante de la construcción de la “maternidad moderna” como planteaba Tarducci (2013), sobre todo porque la progenitora era la “única”, “la mejor dotada” para alimentar a su bebé. La afirmación venía del mundo de lo “racional”, de lo científico, de lo masculino. En oposición radical a posibles consejos del mundo “no racional” y femenino, tal como enfatizaba Turenne (1934, p.18): “No veríamos los desastres con que aún tropezamos, si las futuras madres se hubieran guiado por otros consejos que los de parientes y comadres orgullosas de su animalesca e irracional experiencia sexual y maternal”.

En lo que hace al nacimiento, antes que el Estado se comprometiera con su asistencia, el escenario era heterogéneo y respondía a la clase social. Por lo general, las mujeres de sectores empobrecidos llamaban a parteras o comadronas^4^ para ser atendidas en su domicilio. En Montevideo, si no contaban con dinero para pagar los honorarios o no tenían lugar donde vivir, acudían al Hospital de Caridad. Allí las condiciones de asistencia eran malas, muchas mujeres se contagiaban de infecciones puerperales en su estancia hospitalaria (Turenne, 1909, p.70). Por su parte, las mujeres pobres del sector rural también llamaban a parteras y comadronas o parían sin asistencia, ayudadas por su familia. En los sectores medios y altos los nacimientos se producían en los domicilios con médicos o parteras o en los consultorios de los/as profesionales. Fue el sector que más tardó en hospitalizar el parto.

En 1910 se sancionó la Ley Orgánica de la Asistencia Pública Nacional, a partir de la cual “todo individuo indigente o privado de recursos tiene derecho a la asistencia gratuita por cuenta del Estado” (Uruguay, 1913, p.7). En el inciso “e” del segundo artículo se definió la asistencia y protección de embarazadas y parturientas (p.7).^5^ Con la promulgación de esta ley, se inauguraba una nueva era para la asistencia del parto: el Estado transformaba una instancia que había transcurrido en el marco de la vida privada, en un asunto de política pública cada vez más medicalizado. Entiendo aquí a la medicalización del parto como el proceso por el cual el control y la asistencia del embarazo, el trabajo de parto y el puerperio pasaron a organizarse en función de lo que fue recomendando por el discurso médico. Esto incluye desde erigirse como el saber idóneo para orientar la política pública, legitimarse como el conocimiento autorizado para formar tanto gineco-obstetras como parteras, la incorporación de los resultados de la ciencia obstétrica en la asistencia, la vigilancia de las prácticas de la partería y el crecimiento de la asistencia médica en todo tipo de partos. En este proceso fue central la inauguración de la Casa de Maternidad en 1915.

En el modelo inicial, la asistencia técnica de la Casa de Maternidad se desarrollaba principalmente en el domicilio de las mujeres, atendidas por parteras que se dividían la capital del país en segmentos territoriales. La pernoctación en la sala dormitorio se reservaba para mujeres “desvalidas o abandonadas, cualquiera sea su estado de salud y la edad de su embarazo, y las protegidas que durante el embarazo, el parto o el postparto presenten complicaciones cuyo tratamiento sea imposible efectuar correctamente en su domicilio” (Uruguay, 1916, p.13). Aquella primera política pública, no se circunscribió a la asistencia técnica del embarazo y parto. Fue concebida como una organización para proteger de forma moral, social y legal a las mujeres embarazadas más pobres (Turnes, 2014, p.469; Collazo, Palumbo, Sosa, 2012, p.368). Al instaurar este tipo de protecciones lo que se buscaba era prevenir la degradación humana. Justamente, otro de los problemas inquietantes para la sociedad de principios del siglo XX fue la aparente degeneración de la especie, devenida fundamentalmente de la reproducción de los “ineptos” y de los estragos que producían los tres males del momento: la sífilis, la tuberculosis y el alcoholismo. Científicos, feministas y políticos de un amplio espectro ideológico se comprometieron con la idea de mejorar la humanidad a partir del seguimiento de los preceptos de la eugenesia (eu “bien” y genesia “nacer”) (Sapriza, 2001). Uruguay se alineó a la perspectiva mayoritaria en América Latina, que, a diferencia de países europeos, fue menos radical y no aplicó las medidas más extremas, como la esterilización obligatoria de personas consideradas no aptas para reproducirse (Vásquez, 2011, p.27). Los eugenistas uruguayos se orientaron más hacia el progresismo, muestra de ello fue que en general prefirieron el término especie al de raza (Barrán, 1999). En 1935, la eugenesia ya estaba institucionalizada en el país, existían consultorios que recibían a las personas que querían casarse para hacerles una investigación física, mental y moral, para definir si merecían o no el certificado prenupcial (Sapriza, 2001; Birn, 2019).

La eugenesia de Augusto Turenne (1929, p.27) apuntaba a lograr “generaciones felices, no por el número sino por la calidad”, lo cual lo llevó a promover la anticoncepción en mujeres de sectores empobrecidos. En su discurso de inauguración de la Casa de Maternidad, señaló el deber de la política pública de prevenir los males que podía causar una “madre desvalida a su futuro hijo por el vicio del alcohol, la prostitución y la miseria, engendrando a un ser degenerado” (Collazo, Palumbo, Sosa, 2012, p.269). Concordante con esto, la asistencia de la maternidad de las mujeres pobres rebasará la atención obstétrica e implicará apoyos económicos y sociales para que pudieran mejorar en algo el “nivel cultural y moral”. Los poderes políticos y médicos de la época le dieron relevancia a la capacidad reproductiva de las mujeres (Bock, Thane, 1996), pero desde un enfoque de maternalización (Ledesma, 2015). Para convertirse en “madres” era necesario que siguieran ciertas pautas morales que las alejaran de conductas que pudieran dañar la creación de seres sanos y con ello perjudicar a la nación.

En paralelo, se fueron fortaleciendo las capacidades de los médicos para atender lo maternal, proceso que se consolidó con la creación de la ginecotocología. Los primeros estudiantes de la Facultad de Medicina que hicieron sus prácticas en el Hospital de Caridad no tuvieron la oportunidad de formarse en salas de mujeres. La cátedra de Enfermedades de las Mujeres y los Niños, inaugurada en 1882, fue solo teórica (Pou Ferrari, Pons, 2012, p.111). Es recién en la última década del siglo XIX, cuando la creciente preocupación por la salud fue venciendo al pudor sexual y los médicos acrecentaron su relación con la ginecología y la obstetricia (Barrán, 2008, p.324-325).

En 1900 se fundó la Cátedra de Ginecología y Obstetricia, que hasta 1913 la dictó Augusto Turenne, luego Juan Pou Orfila, de 1913 a 1915. Tanto Augusto Turenne como Juan Pou Orfila escribieron artículos, libros y pronunciaron conferencias, orientando sobre las características y competencias de los médicos que se dedicarían a la nueva especialidad. La disciplina uniría a la ginecología – que trataba las enfermedades del aparato genital de las mujeres en todas sus etapas vitales – con la obstetricia o tocología – que atendía el período de embarazo, parto y puerperio. Esto implicaba que los aspectos ginecológicos y obstétricos se concibieran como inseparables. Para Pou Orfila (1933, 1944) era importante considerar las consecuencias obstétricas de un hecho ginecológico tanto como las consecuencias ginecológicas de un hecho obstétrico. De este modo se prevendrían prácticas que pusieran en riesgo la capacidad de procreación de las mujeres, preocupación encuadrada en los procesos de maternalización, de mejora de la especie y de modernización de la sociedad.

Según Turenne, el antiguo ginecólogo-cirujano no tenía mentalidad obstétrica y, por tanto, realizaba operaciones peligrosas para el porvenir procreacional femenino. “Desde su nacimiento trae la mujer condicionado su destino biológico; a que este se cumpla total e integralmente en todas las etapas de su vida debe el ginecotocólogo dedicar toda su Ciencia para que el ciclo natural de su existencia evolucione dentro de la mayor normalidad” (Turenne, 1940a, p.9). Vemos así cómo la maternalización de las mujeres coincidió históricamente con el proyecto de medicalización de la reproducción biológica, donde la ginecología obstétrica se volvió central (Nari, 2004, p.101-111).

Los obstetras debían adquirir preparación quirúrgica y fisiopatológica, para prevenir ciertas prácticas tradicionales que comenzaban a ser consideradas peligrosas para la vida de las mujeres y, sobre todo, para la “viabilidad fetal” (Turenne, 1940a, p.9).

En el proceso de expansión de la especialidad fue muy importante la creación de maternidades en las principales ciudades del país, conforme avanzaba el siglo fueron instalando salas y pabellones para atender los nacimientos (Collazo, Palumbo, Sosa, 2012, p.351-359). A su vez, las mujeres trabajadoras comenzaron a acceder a derechos para atender su salud reproductiva, pues se crearon seguros de maternidad con derecho a hospitalización. De a poco, el hospital se fue constituyendo en el lugar en donde los médicos sintieron que podían dar las mayores garantías a parturientas y recién nacidos (Pou Ferrari, 2005, p.114). A medida que el parto se asistía cada vez más en hospitales, fue creciendo la investigación obstétrica, la docencia y la enseñanza a futuros ginecotocólogos.

## Intervencionismo obstétrico

El grado de intervención médica en el parto ha sido un asunto muy discutido por la medicina. En el siglo XIX hubo diversidad de corrientes, desde la francesa – que promovía el intervencionismo, con intervención en todos los partos – hasta la escuela de Viena, que proponía la observación paciente, casi sin intervención.

En el Río de la Plata, el grado y la forma de la intervención médica también formó parte de las preocupaciones de los referentes de la ginecotocología naciente. Augusto Turenne, Juan Pou Orfila y Alberto Peralta se declararon en contra de lo que llamaron el intervencionismo de los antiguos médicos parteros. Cuestionaron a la obstetricia clásica, aquella forma de asistencia que practicaba maniobras como la embriotomía (intervención que consiste en seccionar o triturar al feto muerto para disminuir su volumen), la versión (intervención para acomodar la presentación fetal dentro del útero), el parto forzado y el fórceps en versión cefalotripsia (práctica que consiste en aplastar de forma mecánica la cabeza de un feto para extraerlo del útero). Alberto Peralta (1920, p.585), en una conferencia ofrecida en 1920 en la Casa de Maternidad, planteaba: “El médico general en su rol de partero es mucho más peligroso que las mismas parteras en razón de la precipitación con que a menudo recurre a la intervención operatoria”. Se observa así la puja médica por la atención del parto. Los nuevos especialistas apostaban a mostrar la idoneidad de la ginecotocología en la atención del parto. Entendían que la obstetricia clásica perdía tiempo antes de intervenir y, cuando lo hacía, aplicaba técnicas dañinas que podrían haberse sustituido con una operación quirúrgica a tiempo. Agusto Turenne publicó varios artículos científicos en donde comparaba los resultados de la obstetricia clásica con lo que llamó la obstetricia moderna. En los artículos enfatizaba los peligros para el feto de haber optado por la vía clásica. Recordemos que la perspectiva eugenista fue un elemento central en la orientación de la medicina en las décadas en donde se afianzó la ginecotocología. En ese marco, comienzan a aparecer en escena los derechos de embriones y fetos “robustecidos por el advenimiento de métodos y procedimientos terapéuticos” (Turenne, 1919, p.748). En concordancia con esta frase, la obstetricia moderna impulsó un cambio de paradigma en relación a qué vidas proteger. La postura médica de salvar a la madre frente al feto, que orientó a la práctica médica decimonónica, comenzaba a resquebrajarse. Era posible tener en cuenta procedimientos que con cierto riesgo sobre las mujeres viabilizaran la salud fetal. Decía Turenne (1938, p.113-114): “No es nada más que un prejuicio, el de que la vida del niño vale menos que la de la madre. Valdrá menos socialmente, pero tiene un valor ético mayor que el valor social, y el médico no tiene jamás el derecho de hacerlo desaparecer, sin muy fundadas y poderosas razones antes de su vitabilidad”.

## La obstetricia quirúrgica y sus intervenciones: cesárea y episiotomía

Los médicos referentes de la obstetricia moderna recomendaron ejercer desde la noción de obstetricia conservadora o expectante, la cual implicaba que el profesional interviniera lo menos posible para “dejar obrar a la naturaleza en la mayoría de los casos, no perturbarla en general, ayudarla cuando es insuficiente, reemplazarla cuando es ineficaz” (Peralta, 1920, p.582). Esta forma de asistir implicaba disponer de tiempo para acompañar los procesos fisiológicos de las mujeres. Sin embargo, en los documentos y conferencias se observan discusiones sobre si era posible o no, esperar ese tiempo. Varios se manifestaban partidarios de sustituir la espera por la intervención. Augusto Turenne (1930), quien pretendía desmitificar la idea de que la paciencia fuera la mayor virtud de los obstetras, proponía que el médico tuviera una práctica intermitente, dejando a la mujer en trabajo de parto al cuidado de una partera, que llamaría al médico en el momento expulsivo.

Lo planteado sobre el tiempo nos da elementos para observar las tensiones de la ginecotocología para sostener en la práctica lo que postulaba como modelo a seguir. En momentos de su fundación, apuesta teóricamente por un modelo lo menos intervencionista posible, pero a la vez es consciente de las dificultades para practicarlo.

Alberto Peralta y Agusto Turenne fueron entusiastas de lo que llamaron “la época quirúrgica de la obstetricia”, en vez de utilizar fórceps para realizar un “parto forzado”, entendían que se conseguían mejores resultados con una episiotomía (incisión quirúrgica de la vagina y el periné para ampliar el canal del parto) o una cesárea segmentaria (intervención quirúrgica a través de la pared abdominal y uterina). A similares conclusiones arribó el médico Juan Pou Orfila. Según su punto de vista, lo mejor para un nacimiento era culminar con parto natural o con cesárea, no con un parto forzado. Se mostró partidario de la simplificación y contrario a toda maniobra artificial y complicada que “compitiera con la naturaleza” (Pou Orfila, 1944, p.139). En su libro *Síntesis obstétrica*, opone el parto que no necesita intervención, caracterizado como “natural”, al parto con intervención médica, caracterizado como “arte”. El par naturaleza/cultura, oposición impuesta en el paradigma cognitivo de la modernidad (Quintero, 2005), se expresa en la ginecotocología como la posibilidad médico-científica de dominar la naturaleza de la reproducción.

Augusto Turenne fue un precursor de la episiotomía en todos los partos. Entendía que el mecanismo natural para activar la ampliación del canal de parto exigía un esfuerzo fetal importante, donde “el sufrimiento y hasta la muerte del feto son la culminación de esta lucha en la que el médico contempla con orgullo un periné estéticamente intacto, aunque el feto nazca asfíctico o con lesiones del encéfalo que, si no le matan en la primer semana, dejan con frecuencia residuos que se van a traducir en inferioridades mentales o trastornos motores de proyecciones graves para su vida futura” (Turenne, 1930, p.11). Se interpelaba el énfasis en el cuidado del cuerpo femenino a “costa” de afectar la nueva vida. La representación médica del cuerpo de las mujeres se fue adaptando a la maternalización y eugenesia imperantes. En la producción de la “madre moderna” se avanzaba en nuevos permisos para cortar el cuerpo femenino, un cuerpo maternalizado para facilitar la expulsión fetal y el nacimiento de bebés sanos/as.

La cesárea fue la intervención estrella. Hasta la década del 1920 fue temida por la poca sobrevida de las mujeres. Conforme se fue aplicando la asepsia, se fueron mejorando las transfusiones sanguíneas y generalizando el uso de antibióticos y de anestesias, percibiéndola como más segura. En la década de 1940 se produjo lo que se llamó la “liberalización de la cesárea”, pues pasó de utilizarse de un 2% a un 4% a nivel mundial. Luego, a partir de 1960 se ampliaron sus indicaciones, aumentándose su frecuencia con distinta intensidad según los países (Belitzky, 1986, p.5).

Para Turenne (1937, p.94), la cesárea, a la vez que disminuía la mortalidad materna, hacía recobrar al feto “su valor desdeñado”. Era posible cuidar mejor la nueva vida, a la vez que se garantizaba la sobrevivencia de una mujer, que se convertiría en una madre capaz de cuidarla. Según su discurso, la aceptación por parte de las mujeres de las nuevas prácticas obstétricas fue contundente. En sus palabras: “Una sola vez tropecé con una madre que de primeras me dijo que no estaba dispuesta a comprometer su vida por la de su hijo. Pocos días después cedió a mis razonamientos y aceptó la intervención, que afortunadamente no tuve que practicar porque la prueba del parto fue favorable” (Turenne, 1937, p.168). Si bien el hecho de parir no convierte a una mujer en madre, Turenne concibió a la mujer embarazada como una madre y al feto como a su hijo. A su vez, la negación de esta mujer de someterse a una práctica que ponía en riesgo su vida, para salvar a un feto, fue cuestionada por la medicina. La autonomía reproductiva de las mujeres, expandida en tanto fueron accediendo a prácticas obstétricas más seguras, se tensionaba por el mandato maternalista de someterse al parecer médico para hacer lo máximo por el cuidado de la salud fetal.

## La polisemia del parto: el parto normal y sus formas de atención

Parto normal, parto natural, parto fisiológico, parto médico, parto vigilado, parto vaginal, parto conducido, parto intervenido, parto dirigido, parto artificial, parto desviado, parto quirúrgico.

Las distintas formas de adjetivar al parto que utilizó el discurso médico dan cuenta del momento fermental en términos de experimentación clínica y científica. Se discutía en qué tipos de partos tenía sentido aplicar o no tal o cual intervención. En los distintos modos de conceptualizar las fases del trabajo de parto se aprecia el uso del lenguaje mecanicista que señalaba Emily Martin (2006). Al concebir al útero como el motor que podía llevar el “producto” hacia adelante, donde la “buena pelvis” aparece comparada con un riel, “el parto es una complejidad mecánica: hay desplazamiento y progresión. Hay, pues, un objeto pasajero, una distancia transitiva y una fuerza impulsora” (Pou Orfila, 1944, p.29). La mirada mecanicista del cuerpo reproductivo ha tenido consecuencias sobre la relación médico/paciente en la asistencia del parto. Una es la producción de situaciones en donde las mujeres no son tratadas como personas, sino como “objetos” a ser manipulados. Objetos concebidos en tanto cuerpos maternalizados para viabilizar la nueva vida. Si la paciente es un objeto, no se tendrán en cuenta su opinión y sentimientos, la posibilidad de contar con ella intelectualmente será desechada (Davis-Floyd, John, 2004).

Por otra parte, el término de parto normal tuvo un lugar importante en las discusiones analizadas. Aparece definido como un parto que se hacía “por las vías naturales” (Turenne, Iruleguy, 1932, p.13), en ocasiones se utilizaba como sinónimo de “parto fisiológico”. Debía tener una evolución fisiológica y espontánea con la expulsión de un feto vivo y a término. Para varios médicos, tal tipo de parto no ameritaría ninguna intervención, solo la vigilancia (Turenne, 1939, p.8). Pero a la vez dejaron en claro que todo parto aparentemente normal podía transformarse en patológico. De allí la dificultad de una definición que implicaba cierta retrospectiva, nunca se sabía cómo podía terminar un nacimiento.

Augusto Turenne advirtió sobre esto. Opinaba que si bien la definición del parto normal era fácil, no lo era su aplicación. En sus palabras, “no es cosa sencilla establecer con exactitud en qué consiste el parto normal. Prueba de ello son los innumerables ejemplos de partos calificados de tales y que han desembocado en las más temibles distocias, o bien infundadamente han sido objeto de las más absurdas intervenciones” (Turenne, 1939, p.68). La dificultad de establecer qué era un parto normal y qué era un parto patológico fue parte de los fundamentos del crecimiento de la asistencia médica de todos los partos, lo que es parte del proceso – documentado por los estudios de la medicalización de las mujeres – de la patologización del cuerpo femenino. Lo que Meloni (2015, p.24) interpretaba como “la potestad de la medicina de transformar eventos fisiológicos tales como la menstruación o el embarazo y parto en enfermedades”. En los discursos que estoy analizando, sobre todo en los artículos que discuten casos clínicos, no es mayoritaria la idea de enfermedad para definir al embarazo y parto. Más bien lo contrario, como vimos, se inclinaban por una asistencia expectante que confiara en que la gran mayoría de los partos no necesitaba más que vigilancia. No obstante esto, en esos mismos artículos tienden a referirse a las mujeres embarazadas o en proceso de parto con la palabra “enferma”, sin diferenciar necesariamente si había o no patología.

Cabe suponer una diferencia entre el discurso de los catedráticos, que teóricamente se distanciaban del parto como enfermedad, con lo que sucedía en la práctica médica común. Si bien parece fácil la definición teórica, de que en un parto no patológico lo único que cabía era la vigilancia, mucho más difícil habrá sido en la clínica establecer los límites de lo “normal”. La concepción médica de las mujeres como “objetos corpóreos maternalizados” dio mayor legitimidad para intervenir, para patologizar los procesos reproductivos de las mujeres. De allí que en 1941, en el primer número de la *Revista Oficial de la Asociación Obstétrica del Uruguay*^6^ las parteras se rebelaran contra la patologización y publicaran un artículo titulado “La embarazada no es una enferma” (Forno, 1941, p.6).

Otro elemento relacionado con lo anterior tiene que ver con el tiempo. ¿En cuánto tiempo debía progresar un parto “normal”? ¿En qué momento y bajo qué circunstancias era necesario dejar de vigilar e introducir algunas maniobras o intervenciones médicas para hacerlo más dinámico?

En esta discusión aparecía el concepto de “parto médico”. El referente de este tipo de parto fue el doctor Kreis de la escuela de Estrasburgo, quien afirmaba que la mayoría de las mujeres transitaban su parto de forma anormal y por tanto, casi todos los partos debían ser intervenidos por la medicina. Con la intención de anticiparse a las complicaciones, desarrollaba una terapéutica que creaba la necesidad de la intervención médica. Para el médico Alberto Peralta (1933, p.74), en los casos de “parto prolongado” el “parto médico” era la mejor terapéutica. Planteaba que todo parto exigía vigilancia permanente del útero y tratamiento apropiado y oportuno de sus anomalías. El médico argentino dio indicaciones de cómo realizar la rotura artificial y precoz de la bolsa en casos de dinámicas de parto no ajustadas a lo que consideraban normal. Según su casuística, la rotura de bolsa mejoraba la calidad de las contracciones y acortaba la duración del parto.

Hubo consenso en que un “parto médico” no podía “abandonarse a una partera” (Turenne, 1939, p.53). En las fuentes, se relatan casos en que partos normales que fueron asistidos rompiendo membranas, con oxitócicos y demás, concluían con malos resultados cuando los médicos se habían retirado y dejado a parteras en su lugar. Los partidarios del “parto médico” para todos los partos consideraban que las parteras no tenían lugar, lo que abonaba a la exclusión de la partería como profesión idónea en la asistencia al parto.

Augusto Turenne cuestionó la generalización del “parto médico”, tampoco compartía tal denominación. Prefería la noción de “parto conducido” para aquellos casos en que se requería la intervención médica. Según su punto de vista, los defensores del “parto médico” se equivocaban sobre la supuesta excepcionalidad del “parto normal”. Contrastaba con sus estadísticas en donde los casos de trastornos de la dinámica uterina eran mínimos. Según este médico, los defensores del “parto médico” arribaban a tales conclusiones por los efectos de los múltiples exámenes que les hacían a las mujeres, desembocando en agentes de excitación anormal. Llamaba la atención también sobre la infección puerperal que podría acarrear romper bolsas amnióticas y hacer muchos tactos. Según su punto de vista, en las repetidas exploraciones vaginales en busca de patologías, estaba justamente la causa oculta de esa misma patología (Turenne, 1939, p.61). En un círculo vicioso, las intervenciones realizadas con el fin de optimizar el rendimiento del cuerpo gestante maternalizado creaban la necesidad de nuevas intervenciones para corregir lo que la propia medicina había precipitado.

Si bien las parteras agremiadas denunciaron lo que consideraron el arrebato de la asistencia del “parto normal”, desde el discurso médico no se percibieron conflictos en torno a la asistencia médica de todos los partos. Lejos de ello, se autoproclamaron como la profesión especializada en su asistencia, sin distinción de tipo de parto.

En suma, la ginecotocología rechazó las prácticas intervencionistas de la obstetricia clásica – que ponían en riesgo al feto –, pero asumió nuevas versiones del intervencionismo moderno. Según sus discursos, las nuevas prácticas quirúrgicas no acarreaban malos resultados obstétricos, siempre y cuando fueran realizadas por un ginecotocólogo. Las consecuencias, que no revestían carácter mortal, recaerían sobre los cuerpos de las mujeres (cortados, acelerados en las contracciones) y sobre la paulatina exclusión y subalternidad de la partería. Desde la creación de la especialidad en ginecotocología, los médicos no se circunscribieron a la asistencia del parto patológico. Turenne (1938, p.114) recomendó a los médicos jóvenes dedicarse a la asistencia de los partos normales. Lo encontraba ventajoso para las mujeres y provechoso económicamente para ellos.

## Cuerpo, psiquis y maternalización

La teoría social del cuerpo plantea que es posible tomar al cuerpo como objeto de estudio para acceder al análisis de las relaciones entre sujeto y sociedad, así como entre naturaleza y cultura (Esteban, 2004). En los discursos analizados, se registra la creencia de la ginecotocología, de la fuerte influencia del sistema reproductivo en la psiquis y en los comportamientos sociales de las mujeres. La medicina las concebía débiles biológica y emocionalmente, con competencias acotadas para la toma de decisiones. Al decir de Nari (2004, p.109), “la vida social de las mujeres se leyó a partir de su cuerpo”. Debilidades que tenían diferencias según la clase social y pertenencia étnico racial. La mujer débil y dominada halló su expresión más radical en las clases medias (Barrán, 2008, p.339), siendo común el reposo en el embarazo, mientras las mujeres trabajadoras tardaron años en acceder al seguro de maternidad, que les garantizara poder parar de trabajar unos días antes del parto.

El médico Pou Orfila aludía a la ley del triple fundamento – vulnerabilidad, afectabilidad, pansexualismo (VAP) – para referirse a la mentalidad de las mujeres. En sus palabras, “la siquis femenina se caracteriza por la influencia que en ella ejercen las lesiones que acompañan a las funciones sexuales (menstruación, gestación, parturición, lactancia), por la mayor sensibilidad y ternura de su mentalidad, y por el hecho de encontrarse constantemente impregnada del factor sexual, que en el hombre constituye más bien un episodio accidental que un estado permanente” (Pou Orfila, 1933, p.421). Nótese que utiliza el término lesión para caracterizar procesos fisiológicos que enternecerían al cerebro femenino. Y que las concibe inundadas por el factor sexual, no así a los hombres. Tal como ha sido planteado por varias autoras (Rohden, 2001; Nari, 2004), desde finales del siglo XIX, la ginecología puede leerse como una ciencia de la feminidad y de la diferencia sexual. Se partía del supuesto de que el útero hacía de la mujer un “ser especial”. Augusto Turenne (1922, p.409) también sostuvo que el aparato sexual influía en la vida moral y física de las mujeres. Tal era el convencimiento, que en los artículos de época se discutía lo que se llamó “shock obstétrico”, un evento provocado por la extrema sensibilidad nerviosa de la zona genital. Cualquier estímulo sensorial (por ejemplo, un tacto vaginal) podía llevar a una descompensación generalizada de las mujeres.

Por su parte, los hombres, desligados de las tiranías de las hormonas sexuales y sin que su cuerpo fuera un obstáculo para la “racionalidad”, eran construidos como los seres ideales para la vida pública. Otra vez, se observa la influencia de la dicotomía jerarquizante de naturaleza-cultura orientando la organización social de los géneros. Si el “varón racional” se dedicaba a conducir el destino de las sociedades y a estudiar las leyes naturales, la “mujer corporal” se encargaría de la creación y cuidado de los hijos, transformando la potencia de gestar femenina en un atributo maternal y moral de la familia (Martins, 2004, p.264).

### Relación medicina/mujeres

En los discursos analizados aparece la preocupación de los médicos sobre el tipo de relación entre ellos y las mujeres asistidas. Se percibe una insistencia en que los ginecotocólogos lograran entender la personalidad de sus “pacientes” y actuar en consecuencia. Y es que la interacción social sigue patrones que varían de acuerdo a las características de las instituciones y sus contextos, así como de los fines que persiguen los actores (Castro, Erviti, 2015, p.44). En este análisis, entiendo que el interés médico por la construcción de una cierta relación con las mujeres, se sostuvo en la necesidad de crear mejores condiciones para la extensión de la medicalización en el campo reproductivo. Si las mujeres no confiaban en los médicos – como sí lo habían hecho históricamente con otras mujeres, comadronas, parteras y sanadoras –, no seguirían los preceptos higiénicos, eugénicos y de la puericultura que las transformarían en mejores madres. Los médicos tenían que construir una confianza devenida de la ciencia, una ciencia consciente (en ese momento) de su incapacidad técnica, para lidiar con muchas de las afecciones que pretendía tratar. De allí la recomendación de Pou Orfila (1933, p.422) de tratamientos que abarcaran la totalidad de la persona, física y moral: “Al aplicar muchos de estos tratamientos habrá que colocarse más bien en un terreno práctico y empírico, renunciando, en cierto modo, al concepto determinista o causal, científicamente riguroso, que el estado actual de nuestros conocimientos no permite todavía aplicar”. Para Agusto Turenne (1938, p.108), la autoridad del médico no derivaba solo de su capacidad técnica, sino más bien de su cultura. Por cultura se estaba refiriendo a una educación vasta que le permitiera amoldarse a cada situación, colocarse en el mejor nivel para comunicarse con cada paciente y escoger los argumentos para reforzar su autoridad moral.

Las recomendaciones de Augusto Turenne para el caso del parto sin dolor nos brindan una oportunidad de visualizar el énfasis en la maternalización que aplicó la medicina para lograr la obediencia de las mujeres en situaciones obstétricas.

En Uruguay, hasta la década de 1940, las discusiones médicas en torno a los pro y los contra de disminuir los dolores de parto giraron en torno al uso de distintas drogas, la analgesia se concebía solo desde lo medicamentoso. En 1938, en la lección inaugural para la toma de posesión de la cátedra de obstetricia y ginecología de la Facultad de Medicina, el doctor Manuel Rodríguez (1938, p.1053) afirmaba: “El parto sin dolor empieza a ser una de las más bellas conquistas con que la obstetricia premia a las mujeres que sienten en el alma el sublime anhelo de ser madre”. En esta frase hay una apuesta doble: a la maternalización a través de una recompensa para las mujeres que trajeran hijos al mundo y a la victoria de la “cultura” médica sobre la “naturaleza”, al mitigar la sensación que ha definido históricamente al parto en sociedades cristianas: el dolor.

Por su parte, Agusto Turenne (1940b) dictó una conferencia dedicada al dolor en el parto y las terapéuticas para disminuirlo. En el discurso relató una situación que presenció en París: el suicidio de una mujer por los dolores de un parto de caderas. Trayendo este relato, planteaba la necesidad de que la medicina se comprometiera con el alivio del sufrimiento materno. En la conferencia, dio cuenta de su experiencia con varias sustancias analgésicas y cuestionó el uso de algunas porque acarreaban consecuencias negativas para el feto y la mujer. Planteaba que en ese momento no existía parto sin dolor y sin peligro, todas las analgesias tenían posibilidades de causar daño. Mitigar el dolor o no resultaba una decisión compleja para la medicina, que incluía valorar el tipo de relación médico/paciente logrado en el correr de la atención del embarazo. Al médico le preocupaba cómo crear una relación con las mujeres que garantizara la máxima aceptación de los consejos médicos. Partía de la base de que la “grávida” era “siempre una inestable emocional” y muchas veces frágil biológicamente. Ante esto, los médicos debían aprovechar los nueve meses de embarazo para “apoderarse de la psiquis de la grávida”, de modo de realizar “una constante acción de sugestión” para infundirles optimismo frente al parto (Turenne, 1940b, p.13). Si la mujer exigía un parto con anesyesia, y el médico entendía que no era lo mejor, se tendría que recurrir a tal sugestión. Si esto no alcanzaba, había que apelar al “instinto maternal”, si es que esa mujer daba muestras de tenerlo. En esos casos, se la debía persuadir con el riesgo para la vida de su hijo al aplicar una analgesia. Según palabras de Turenne (1940b, p.14), el problema serio se les presentaba cuando entendían que las mujeres tenían “amenguado el sentimiento maternal”, en esas situaciones era muy difícil convencerlas de un parto sin anestesia. Allí, el médico debía “utilizar toda su autoridad moral para no transformarse en juguete de mujeres de mentalidad desviada o pervertida”. Se desplegaba así una categorización de las mujeres en función de su “capacidad maternal” y su correlato en el tratamiento médico. El mito social de mujer = madre con un “instinto materno” que la haría velar por sus hijos e hijas (Fernández, 1994, p.169) no parecía ser algo dado. Los médicos tuvieron que sopesar el “espíritu maternal” y su funcionalidad para acatar su consejo.

En síntesis, en las discusiones y terapéuticas sobre el parto sin dolor, se expresa con claridad la relación entre las intervenciones obstétricas y la maternalización. Quizás algunas mujeres preferían un cierto riesgo para el hijo con tal de pasar menos dolor, pero la medicina apeló a un arsenal de estrategias para convencer a las “madres modernas” de hacer lo que fuera para que su hijo o hija no pasara por ningún riesgo.

## Consideraciones finales

El proceso de especialización de la ginecotocología conllevó grandes cambios en las prácticas obstétricas. Las transformaciones se introdujeron con el fundamento de mejorar los resultados obstétricos, en especial para cuidar al feto de daños producidos por el trabajo de parto y por las maniobras de la obstetricia clásica, que pasaron a evaluarse como obsoletas y peligrosas.

En el proceso de consolidación de la especialidad médica se puso en juego la noción de intervencionismo obstétrico. Los referentes de la ginecotocología se quisieron diferenciar de los médicos parteros anteriores, los nuevos expertos cuestionaron la desvalorización de la vida del feto frente a la de la mujer. Introdujeron una visión médica que transformó las prácticas, instalando la episiotomía y la cesárea como intervenciones que mejoraban los resultados obstétricos. Si en cada momento histórico se pueden leer las relaciones de género en la construcción del cuerpo, aquel cuerpo reproductivo se comienza a simbolizar como material a ser cortado – con el cuidado de las nuevas técnicas quirúrgicas – para mejorar la salud fetal.

Si bien la obstetricia moderna cuestionó el intervencionismo de sus predecesores, tuvo problemas para aplicar un modelo menos intervencionista. Se daban cuenta que el intervencionismo no fue mitigado por la sofisticación de las nuevas técnicas operatorias. Las intervenciones fueron cambiando, las prácticas consideradas peligrosas para el feto tendieron a bajar, pero aumentaban otras. Desde el comienzo de la especialidad fueron conscientes de sus dificultades para asistir de forma adecuada. En particular, manifestaron que no contaban con las condiciones – de formación y de tipo de ejercicio profesional – para esperar el tiempo necesario de los procesos fisiológicos del parto, lo que los llevaba a intervenir para no esperar.

Por otra parte, a partir de analizar las discusiones sobre el parto normal, se puede observar la progresiva apropiación de la medicina para asistir todos los nacimientos. Los médicos utilizaron el argumento de la dificultad para definir qué era un parto normal y qué era un parto patológico como fundamento para la asistencia médica de todos los partos. Extendiéndose así, la reducción de la partería en la asistencia y creciendo su subordinación técnica.

La relación de la medicina con las mujeres estuvo marcada por la maternalización y por un accionar manipulador sobre la base de controlar la información en torno a los daños fetales y las intervenciones propuestas. Apelaron a la volición de las mujeres de cuidar al feto para generar mejores condiciones para la aceptación del mandato médico. Según la “capacidad maternal”, las mujeres responderían más o menos a sus mandatos. En todo caso, los catedráticos advirtieron de cuidarse de no ser manipulados por mujeres “pervertidas” definidas como las que no querían pasar por los dolores del parto sin importar las consecuencias fetales. Para los médicos, la capacidad maternal, homologada al sacrificio por el hijo, era una dimensión muy influyente en la posibilidad de parir con dolor y no exigir anestesias. De este modo es posible conectar la maternalización de las mujeres con las intervenciones obstétricas. Una mujer igualada a una madre se sometería a la intervención médica por no perjudicar a su bebé.

## NOTAS

1 En el siglo XIX en Francia, se fundaron los primeros Consultorios Gota de Leche, los cuales examinaban y pesaban a los lactantes, asistían a los enfermos, proporcionaban alimentación artificial, esterilizaban la leche y enseñaban puericultura a las mujeres madres (Turnes, 2014). En Uruguay, el primero se fundó en Montevideo, en el año 1907, bajo la dirección del médico Julio Bauzá (Uruguay, 1913).

2 Dicho término fue inventado en Uruguay. En otros países suele utilizarse ginecobstetricia o ginecología y obstetricia (Pou Ferrari, Pons, 2012, p.128).

3 El torno era un instrumento creado para facilitar el abandono anónimo de niños y niñas. Consistía en una cesta giratoria colocada en una pared de hospitales y asilos. Cuando se dejaba un recién nacido se hacía sonar una campana. La persona que hacía guardia en el interior del recinto giraba el torno y recogía al bebé.

4 En este trabajo se concibe a las parteras tituladas o profesionales, a personas que estudiaron partería y se titularon en instituciones reconocidas en su país. Mientras que por comadrona nos estamos refiriendo a mujeres que aprendieron el oficio de partear de forma empírica y no se sometieron a ningún examen para titularse.

5 Art. 2º La Asistencia Pública Nacional tendrá a su cargo la organización y establecimiento y servicios destinados a atender las siguientes necesidades sociales: (1) asistencia de enfermos; (2) asistencia y cuidado de alienados; (3) asistencia y protección de ancianos desamparados; inválidos y crónicos; (4) asistencia y tutela de niños desamparados; (5) asistencia y protección de embarazadas y parturientas; (6) protección a la infancia, sin perjuicio a la que corresponde al patronato de menores.

6 Fue una revista publicada por las parteras agremiadas entre 1941 y 1959. Se publicaron 27 números que se encuentran en la Sala General de la Biblioteca Nacional.
